# Organoids to model liver disease

**DOI:** 10.1016/j.jhepr.2020.100198

**Published:** 2020-10-22

**Authors:** Sandro Nuciforo, Markus H. Heim

**Affiliations:** 1 Department of Biomedicine, University Hospital Basel, University of Basel, CH-4031 Basel, Switzerland; 2 Clarunis, University Center for Gastrointestinal and Liver Diseases, CH-4002 Basel, Switzerland

**Keywords:** Liver disease, Disease modelling, Organoids, 3D cultures, Personalised medicine, Preclinical models, A1AT, alpha-1 antitrypsin, ALD, alcohol-related liver disease, CCA, cholangiocarcinoma, CFTR, cystic fibrosis transmembrane conductance regulator, CHC, combined hepato-cholangiocarcinoma, Chol-orgs, cholangiocyte organoids, CLD, chronic liver disease, CTLN1, citrullinemia type 1, EGF, epidermal growth factor, ER, endoplasmic reticulum, ESCs, embryonic stem cells, FFAs, free fatty acids, HCC, hepatocellular carcinoma, Hep-orgs, hepatocyte organoids, HUVEC, human umbilical vein endothelial cells, IL-, interleukin-, iPSC, induced pluripotent stem cell, MSC, mesenchymal stem cell, NAFLD, non-alcoholic fatty liver disease, PDO, patient-derived organoid, PDX, patient-derived xenograft, PHH, primary human hepatocyte, PSC, primary sclerosing cholangitis

## Abstract

The organoid model represents a major breakthrough in cell biology that has revolutionised biomedical research. Organoids are 3D physiological *in vitro* structures that recapitulate morphological and functional features of *in vivo* tissues and offer significant advantages over traditional cell culture methods. Liver organoids are of particular interest because of the pleiotropy of functions exerted by the human liver, their utility to model different liver diseases, and their potential application as cell-based therapies in regenerative medicine. Moreover, because they can be derived from patient tissues, organoid models offer new perspectives in personalised medicine and drug discovery. In this review, we discuss the current liver organoid models for the study of liver disease.

**Key points**

• Current *in vitro* and *in vivo* models of liver diseases such as primary human hepatocytes, cancer cell lines and animal models fail to recapitulate key aspects of human liver biology.
• Liver organoids have emerged as a novel *in vitro* tool for the study of hepatic physiology and pathophysiology.
• Liver organoids can be derived from patient-derived tissue biopsies or pluripotent stem cells and enable the generation of personalised models that preserve inter-individual features.
• Liver organoid systems are suited for translational research and drug development and may find clinical application for regenerative medicine in the near future.

## Introduction

Liver disease is a major global health burden responsible for over 2 million deaths per annum worldwide.^1^ Most liver-related morbidity and mortality is caused by chronic liver disease (CLD). The major risk factors for the development of CLD include HBV/HCV infections, excessive alcohol consumption and non-alcoholic fatty liver disease (NAFLD). The clinical course of CLD is highly variable. Persistent necro-inflammation with concomitant regeneration can cause progressive fibrosis and cirrhosis, and predispose patients to liver cancer. For many patients with CLD, therapeutic options remain limited. Scientific progress in the field is hindered by the lack of suitable animal or cell culture models. Past and current *in vitro* studies mostly relied on cell lines derived from hepatomas and hepatocarcinomas and on primary human hepatocytes (PHHs). PHHs preserve many hepatocyte characteristics and provide faithful *in vitro* models for studying drug metabolism and toxicity, viral infections, and genetic diseases. However, because their viability in culture is restricted to a few days, fresh donor tissue is constantly required. Cell lines derived from hepatomas and hepatocarcinomas offer unlimited proliferation but are of cancerous origin and lack critical features of normal hepatocytes. Recently, organoids emerged as new physiological model systems for the study of various organs and tissues.^2^ Organoid cultures offer many advantages as they can be generated from healthy and diseased tissues, can be expanded over long periods (thus retaining their genetic stability), and can be cryopreserved to generate biobanks (Table 1). Herein, we will discuss different approaches for the generation of liver organoid models and how they can be exploited for the study of liver disease.

**Table 1:** Comparison of different hepatic *in vitro* model systems.

Feature	PHH	iPSCs^a^	Hep-orgs^b^	Chol-orgs^c^	Multilineage organoids^d^	Cancer cell lines	Liver cancer organoids
Success rate of establishment	++	++	+	+++	+	+	+/++
Time to establishment	short	medium	medium	short	long	medium	medium
Long-term maintenance^e^	-	++	+	++	-	+++	+++
Relative cost	$	$$	$$$	$$$	$$$	$	$$$
Morphological complexity & 3D growth	-	+	++	++	+++	-	++
Preservation of genetic background	+++	+++	+++	+++	+++	+	++
Recapitulation of hepatic functions	+++	++	++	++	++	+	+
Genetic stability	n.e.^f^	++	n.a.	++	n.a.	+	++
Genetic manipulation	+	+++	n.a.	+++	+++	+++	+++
High-throughput screening^e^	+	+++	+	++	+	+++	+++

Chol-orgs, cholangiocyte organoids; Hep-orgs, hepatocyte organoids; iPSC, induced pluripotent stem cell; n.a., not assessed; n.e., not evaluable; PHH, primary human hepatocytes.

(-) unsuitable, (+) possible, (++) suitable, (+++) best. Note: when comparing phenotypic and functional properties, we refer to iPSCs, chol-orgs, and multilineage organoids differentiated into hepatocyte-like cells. For all other features we consider undifferentiated cultures in their respective proliferative expansion phase. All patient-derived models are very good at recapitulating the genetic background, however, in case of cancer cell lines and cancer organoids there is additional complexity due to intratumoural genetic heterogeneity that is preserved at different degrees depending on the model. Genetic manipulation for disease modelling is well possible for models derived from iPSCs as well as adult tissues.

a iPSCs derived from fibroblasts and other cellular sources.^34^^,^^35^

b Hep-orgs of adult and foetal origin.[27], [28], [29]

c Chol-orgs derived from adult liver, foetal liver, and pluripotent stem cells.[23], [24], [25], [26]^,^^29^^,^^38^^,^^39^

d Multilineage organoids and liver buds derived from pluripotent stem cells.^36^^,^^37^^,^^44^

e Mainly taking into consideration ease of maintenance and proliferation rate.

f n.e., not evaluable because of lack of proliferation and expansion in culture.

## Organoids: origin and basic concepts

More than a century ago, the first hanging drop culture experiments for the study of organogenesis revealed the intrinsic ability of cells to interact and self-organise into organ-like structures.^3^ Subsequent decades of work on stem cell and extracellular matrix biology followed by continuous improvements and innovations in cell culture technologies — in particular those allowing 3D cell growth — served as a foundation for the development of the organoid culture system. Pioneered in the Clevers lab for the study of intestinal stem cells, the organoid technology is a major advance for the stem cell field as it enables researchers to stably grow self-renewing intestinal epithelia that recapitulate the crypt-villus architecture.^4^ Surprisingly, the culture protocol for the growth of organoids is rather simple, yet builds on knowledge gained from years of work on the intestinal stem cell niche.^5^^,^^6^ As described in the seminal study by Sato and colleagues, single Lgr5-positive stem cells isolated from the small intestinal epithelium are embedded in Matrigel®, an extracellular matrix isolated from Engelbreth-Holm-Swarm tumours, and cultured in a growth factor supplemented medium that recapitulates the signals from the intestinal stem cell niche.^4^ Among the factors included in the medium, such as epidermal growth factor (EGF) and Noggin, the Wnt pathway potentiator R-spondin 1 emerged to be a critical requirement for the expansion, differentiation and self-organisation of the growing small intestinal organoids. In the following years, the protocol for deriving organoid cultures was successfully applied to other organs including the colon, stomach, prostate, liver, fallopian tube, mammary gland, salivary gland, endometrium, placenta, pancreas and lungs, by adapting the culture conditions to recapitulate the niche signals of the corresponding tissues.^7^

Embryonic stem cells (ESCs) and induced pluripotent stem cells (iPSCs) represent an alternative source for growing organoids that has particular importance for tissues with no established protocols for derivation of organoids from adult stem cells, such as the brain, kidney, inner ear, retina and thyroid.^7^

During the past 10 years, the research community has witnessed a burst of studies in the rapidly developing organoid field. The term *organoid* simply means resembling an organ. A more explicit definition proposed by Lancaster and Knoblich is based on 3 essential requirements that need to be fulfilled: first, an organoid must contain more than 1 cell type of the organ it models; second, it must recapitulate some of the specific functions of that organ; and third, the cells should have a similar spatial organisation as the organ.^8^ Since most liver organoid systems are composed of a single cell lineage, the slightly different definition of an organoid proposed by Huch and Koo seems more appropriate.^9^ They define an organoid as a 3D structure in which cells spontaneously self-organise into progenitors and differentiated functional cell types that resemble the original organ and recapitulate some of its functions.^9^ In the meantime, additional definitions that are more or less restrictive have been proposed.^3^

## Liver organoids

### Liver structure and function

The liver performs a multitude of essential functions including biotransformation of endogenous and exogenous metabolites, biosynthesis of plasma proteins, storage of macromolecules such as glycogen and lipids, and production of bile. Six major cell types populate the liver: hepatocytes, large polyhedral epithelial cells and the primary parenchymal cell type in the liver; cholangiocytes, also termed biliary epithelial cells (BECs), that line up the bile ducts; hepatic stellate cells, pericytes storing vitamin A and involved in extracellular matrix biosynthesis upon liver injury; Kupffer cells, liver-resident macrophages; liver sinusoidal endothelial cells, specialised endothelial cells forming the fenestrated lining of the hepatic sinusoid; and portal fibroblasts, residing in the stroma of the portal tract in close proximity to bile ducts.

The liver is organised in hexagonal lobules, with sheets of hepatocytes radiating out a central vein towards 6 portal tracts. Each portal tract comprises terminal branches of the portal vein, hepatic artery and bile ducts. The hepatic artery supplies the liver with oxygenated blood that mixes with portal venous blood and flows along the porto-central axis. The resulting oxygen gradient together with spatially restrained signaling pathway activity (*e.g.* pericentral Wnt signaling and periportal Hedgehog signaling) contribute to the formation of what is termed metabolic zonation: distinct enzymatic activities and cellular functions are spatially zonated along the liver lobule.^10^ The diversity of essential liver functions can be compromised by various chronic liver diseases that impair hepatocyte function and cause a progressive disruption of the lobular architecture resulting in cirrhosis.

### Adult-derived liver organoids

The ability to grow patient-specific mature hepatocytes *in vitro* is an unmet need in translational research related to the study of liver toxicology, virology, metabolic and genetic diseases as well as regenerative medicine. The discovery and *in vitro* culture of Lgr5-positive stem cell of the intestinal crypts marked the beginning of the organoid research field as we know it.^4^^,^^5^ In the following years, additional studies reported the discovery of Lgr5-positive stem cells in many more organs, underlining the importance of Wnt signalling as a master signalling pathway for different stem cell compartments.^11^

In the liver, the existence of a specialised population of stem cells has been controversial. During homeostasis, hepatocytes are terminally differentiated cells that persist for over a year without cell division. However, liver injury following acute or chronic insults such as toxins, viruses, or physical damage results in the activation of a potent proliferative program in hepatocytes and an efficient repopulation of the lost cell pool within a very short timeframe.^12^ The source of regenerating hepatocytes in the liver has been attributed to different cell populations. The first concept of a liver stem cell was based on oval cells, a population of bipotent progenitor cells derived from BECs within the Canal of Hering.[13], [14], [15] Similarly, periportal hepatocytes were reported to regenerate the liver mass following chronic injury.^16^ However, the longstanding concept of a periportal stem cell niche was recently challenged by a study reporting the presence of pericentral Axin2-positive stem cells,^17^ and in turn re-challenged by more recent studies supporting the notion that all hepatocytes along the porto-central axis retain the ability to repopulate the liver during homeostasis or following injury.[18], [19], [20] However, while unlikely to represent hepatic stem cells, pericentral hepatocytes are unique with regard to their persistently high Wnt signaling activity, like intestinal stem cells.^17^^,^^21^ On the other side, activation of Wnt signaling can also be detected in BECs following liver damage.^22^ By using culture conditions similar to those for intestinal organoids, Huch and colleagues reported the first liver organoid system based on the expansion of Lgr5-positive biliary cells isolated from a mouse model of liver injury.^23^ Remarkably, healthy biliary duct fragments isolated from undamaged mouse livers could also be expanded using the same culture protocol.^23^ Subsequently, minor refinements to the culture protocol also enabled the generation of organoids from healthy human liver tissue.^24^ These so-called chol-orgs (cholangiocyte-derived organoids) closely mirror the oval cell-based response to liver damage. Chol-orgs are closely related to cholangiocytes in terms of morphology, marker expression and functional properties.[24], [25], [26] However, compared to primary cholangiocytes, chol-orgs express lower levels of mature markers and increased levels of foetal markers, indicating that the cells are not fully differentiated.^25^^,^^26^ Nevertheless, several functional properties of mature cholangiocytes could be efficiently modeled in chol-orgs, including MDR1-dependent secretory capability, export of bile acids from the organoid lumen, and response to physiological stimuli such as somatostatin, secretin and vascular endothelial growth factor.^25^^,^^26^ Therefore, chol-orgs represent a valuable *in vitro* model that recapitulates key features of the *in vivo* biliary epithelium.

**Fig. 1:**
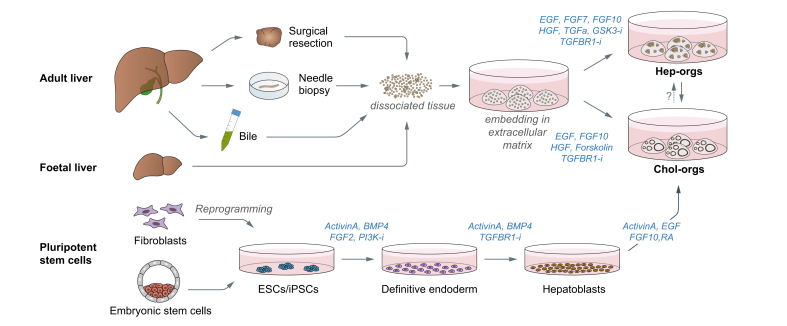
Workflow for the generation of liver-derived organoids. Liver organoids can be derived from different sources such as adult liver tissue, foetal liver tissue, bile and pluripotent stem cells. Adult- and foetal liver tissue-derived progenitor cells can be stimulated to form organoids of the hepatocyte (hep-org) or cholangiocyte (chol-org) lineage following incubation with a defined combination of growth factors.^23^^,^^24^^,^^27^^,^^28^ Foetal liver-derived progenitors/hepatoblasts can give rise to both hep-orgs and chol-orgs.^29^ Progenitor cells isolated from bile can be grown as chol-orgs. Hep-orgs can be partially transdifferentiated into chol-orgs, but not vice-versa. However, chol-orgs can undergo differentiation into hepatocyte- or cholangiocyte-like cells according to Huch and colleagues (not shown).^23^^,^^24^ Pluripotent stem cells of embryonic (ESC) or somatic (iPSC) origin first require a 3-stage differentiation protocol to generate hepatoblast-like cells (depicted here the protocol according to Sampaziotis *et al.*^25^), that are subsequently embedded in extracellular matrix to promote 3D growth and organoid formation. BMP, bone morphogenetic protein; EGF, epidermal growth factor; ESC, embryonic stem cell; FGF, fibroblast growth factor; GSK3-i, glycogen synthase kinase 3 inhibitor; HGF, hepatocyte growth factor; iPSC, induced pluripotent stem cell; PI3K-i, phosphoinositide 3 kinase inhibitor; RA, retinoic acid; TGFα, transforming growth factor alpha; TGFBR1-i, transforming growth factor beta receptor 1 inhibitor.

Very recently, 2 revised versions of the original chol-org culture protocol enabled the growth of organoids from adult primary hepatocytes and foetal liver cells, termed hepatocyte organoids (hep-orgs), providing a tool to model the proliferative response of hepatocytes seen after partial hepatectomy.^27^^,^^28^ Interestingly, a subpopulation of hepatoblasts marked by the expression of Lgr5 can serve as cellular source for the generation of both hep-orgs and chol-orgs, underlining the bipotential plasticity of hepatoblasts.^29^ Both liver-derived organoid models, hep-orgs and chol-orgs, represent distinct entities (Fig. 1), yet their culture protocols share many features. They both require mitogenic signaling through the EGF-, hepatocyte growth factor-, and fibroblast growth factor-receptors and inhibition of TGF-β signaling to allow for long-term expansion. However, individual molecules are added depending on the cell type, forskolin for chol-orgs and GSK3B- and ROCK1 inhibitors for hep-orgs. Potentiation of Wnt signaling by R-spondin 1 is required for human and mouse chol-org, as well as human hep-org, expansion,^23^^,^^24^^,^^27^ but not for mouse hep-org expansion.^28^ Extracellular matrix hydrogels such as Matrigel® or Cultrex® Basement Membrane Extract provide structural support to the growing organoids and enable their 3D suspended growth. Moreover, it is also well recognised that composition and stiffness is crucial for colony formation efficiency, proliferation and differentiation as revealed from studies using artificial matrices.[30], [31], [32] A direct comparison of chol-orgs and hep-orgs revealed 2 critical features that differ significantly between the culture systems, clonogenicity and growth rate.^27^ Nearly every third ductal cell is able to initiate chol-org formation in a process involving major epigenetic and transcriptional remodeling,^24^^,^^33^ while only 1 in a 100 hepatocytes expands and forms hep-orgs.^27^ Chol-orgs proliferate rapidly with doubling times of ∼60 hours for more than 20 passages, whereas hep-orgs proliferate much slower and double every 5–7 days, if derived from foetal livers, or are passaged 1–2 times every 50–75 days if derived from adult livers.^24^^,^^27^

Under expanding conditions, chol-orgs grow as cystic structures composed of bipotent progenitors expressing markers of the hepatocyte and cholangiocyte lineage.^23^^,^^24^ Using a defined differentiation medium, chol-orgs can be further differentiated into functional hepatocyte-like cells (HLCs) that are able to secrete albumin and carry out different liver specific functions including glycogen storage, ammonia and xenobiotic metabolism, uptake of lipids and production of bile acids. However, differentiated chol-orgs display residual expression of ductal markers indicating incomplete hepatic maturation. Complete loss of ductal markers could only be observed following chol-org transplantation *in vivo*.^24^

In contrast, hepatic maturation is far more advanced in hep-org cultures as they more closely resemble PHHs in terms of hepatocyte marker expression and functional properties.^27^ Hep-orgs grow as grape-like structures, unlike cystic chol-orgs, and contain larger cells with a well-defined polygonal shape. Transmission electron microscopy also revealed glycogen particles and structures resembling the bile canalicular network. In a direct comparison, albumin secretion in hep-orgs was comparable to that of PHH, while alpha-1 antitrypsin (A1AT) secretion was remarkably lower in both foetal- and adult liver-derived hep-orgs.^27^ The activity of cytochromes was variable depending on tissue origin, whereby adult liver-derived hep-orgs displayed higher CYP3A4 activity compared to PHH, while foetal hep-orgs were clearly inferior.^27^ In general, in contrast to human hep-orgs, the hepatic phenotype of mouse-derived hep-org cultures seemed to be less mature since the secretion of albumin and the activity of cytochromes was lower than in primary mouse hepatocytes.^27^^,^^28^

Nevertheless, aside from the advantages offered by human hep-org cultures, the fact that the majority were derived from foetal liver cells rather than adult PHHs represents a major limitation of the current hep-org culture protocol, especially owing to the limited availability of foetal livers from human donors. Moreover, the majority of human hep-orgs derived from foetal livers have a greater expansion potential with over 16 passages in culture, while adult liver-derived hep-orgs cease growth after 1–2 passages (2.5 months).^27^ Taken together, hep-orgs are superior to chol-org-derived HLCs for modelling human hepatocytes *in vitro*. However, they are not yet widely used because of the limited success rate in generating and expanding hep-orgs. For most research groups, PHHs therefore still represent the gold-standard *in vitro* hepatocyte model because of their superior phenotypic and functional maturation level compared to hep-orgs, despite their lack of 3D organisation and ability to proliferate. It is however conceivable that future improvements in the culture conditions will allow robust expansion of adult hepatocyte-derived hep-orgs or, alternatively, chol-org-derived HLCs with greater levels of maturation.

### Pluripotent stem cell-derived liver organoids

An alternative route for the generation of *in vitro* liver models involves the use of patient-derived iPSCs. Several research groups proposed a variety of protocols for the stepwise differentiation of iPSCs into HLCs by recapitulating the different stages of embryonic liver development.^34^ Most differentiation protocols consist of 3 main stages, all of them typically beginning with the generation and commitment of iPSCs to the endodermal lineage^34^^,^^35^ (Fig. 1). In a second step, cells with the acquired features of the definitive endoderm are further differentiated into hepatoblasts, before being matured into HLCs in a third step. The advantages of using iPSCs for the derivation of HLCs are similar to those for chol-orgs as both models retain the genetic background of the original patient and the expansion of the cells prior to their differentiation is highly efficient and virtually unlimited.

The success of organoid technology has highlighted the advantages of 3D growth, an aspect that was for a long time neglected in iPSC protocols. Takebe and colleagues were the first to generate an organ-like *in vitro* liver model by co-culturing hepatic endodermal cells derived from iPSCs together with human mesenchymal stem cells (MSCs) and human umbilical vein endothelial cells (HUVECs).^36^ The 3 cell lineages spontaneously self-organised into aggregates termed iPSC-liver buds (iPSC-LBs) that recapitulated several phases of organogenesis including the formation of a vascular-like endothelial network. Strikingly, the vascular networks became functional following transplantation of iPSC-LBs *in vivo*, thereby enabling further maturation of the liver buds that started to metabolise drugs and to secrete albumin into the bloodstream. Moreover, implantation of liver buds in the mesentery improved survival in a mouse model of acute liver failure, providing evidence for the use of iPSC-LBs as a novel source of transplantable tissue for liver regeneration purposes. In a follow-up study, the culture protocol was further refined to enable scalable manufacturing of liver buds entirely from iPSCs, circumventing the need for postnatally derived MSCs and HUVECs.^37^ However, combining multiple cell types in a single co-culture system bears the challenge of selecting the right combination of culture conditions to maintain the different cell types. Following pioneering studies using liver buds, numerous alternative protocols for the generation of liver organoids from pluripotent stem cells have been reported. Most of them combine the classic stepwise differentiation of iPSCs with extracellular matrix-supported 3D growth and a defined modulation of the culture conditions that allows for the generation of liver organoids with single or multiple cell lineages. For example, hepatoblast-like cells could be precisely differentiated into cholangiocyte organoids^25^^,^^26^^,^^38^^,^^39^ or, alternatively, into hepato-biliary organoids with features of both lineages.[40], [41], [42] Similarly, fibroblasts could be also directly reprogrammed into HLCs and then further matured into organoids by cell aggregation cultures, without the need to generate iPSCs.^43^ Moreover, Ouchi and colleagues reported the simultaneous differentiation of foregut endodermal progenitors into tri-lineage liver organoids containing hepatic progenitors, stellate- and Kupffer-like cells.^44^

Compared to earlier models of iPSC-derived hepatic cell cultures, growth in the third dimension clearly enhanced the hepatic maturation potential, which was reflected at the functional and morphological level. However, despite the execution of numerous functions such as Albumin and bile acid production, glycogen synthesis, lipid accumulation, metabolic activity including elimination of ammonia and detoxification reactions, the maturation level of current *in vitro* hepatocyte models is still inferior to PHHs. In addition, most of the available iPSC-derived liver models do not self-renew following differentiation and require additional rounds of *de novo* differentiation starting over from iPSCs. Nevertheless, organoid models, irrespective of their origin, offer an unprecedented means to study liver disease *in vitro*.

## Disease modeling using liver organoids

The possibility of deriving organoid models from patients opens up new opportunities for the study of liver diseases in a truly translational setting. The use of adult tissue samples enables generation of organoids that retain the genetic background of the respective individual including specific disease-causing mutations as in the case of monogenic diseases and cancer. Moreover, the CRISPR-Cas9 technology facilitates the precise introduction and correction of such mutations for the study of their function, pathogenicity and association with targeted therapies.^45^ Hereafter, we discuss current models of liver disease based on organoid culture systems (Table 2).

**Table 2:** Organoid-based models of liver disease.

Disease	Species	Organoid source and derivation	References
Alagille syndrome	Human Mouse	Adult tissue (surgical resection) iPSCs (fibroblast-derived) Adult tissue (GEMM)	Huch *et al.*^24^ Guan *et al.*^40^ Andersson *et al.*^49^
Alcohol-related liver disease	Human	ESCs	Wang *et al.*^67^
Alpha-1 antitrypsin deficiency	Human	Adult tissue (surgical resection; liver transplantation; biopsy)	Huch *et al.*^24^; Gomez-Mariano *et al.*^51^
Citrullinemia type I	Human	iPSCs (fibroblast-derived)	Akbari *et al.*^38^
Cystic fibrosis	Human	ESCs iPSCs (fibroblast-derived; peripheral blood derived)	Ogawa *et al.*^26^ Sampaziotis *et al.*^25^
HBV infection	Human	iPSCs (fibroblast-derived)	Nie *et al.*^95^
Primary liver cancer	Human Mouse	Adult tissue (surgical resection) Adult tissue (needle biopsy) Adult tissue (liver transplantation; genome editing) Adult tissue (chemical carcinogenesis) Adult tissue (GEMM)	Broutier *et al.*^81^; Li *et al.*^89^ Nuciforo *et al.*^82^ Artegiani *et al.*^91^ Cao *et al.*^83^ Saborowski *et al.*^92^
Primary sclerosing cholangitis	Human	Adult tissue (bile-derived; surgical resection)	Soroka *et al.*^94^
Steatosis, steatohepatitis	Human Cat	iPSCs (fibroblast-derived) Adult tissue (post-mortem)	Ouchi *et al.*^44^ Kruitwagen *et al.*^71^; Haaker *et al.*^72^
Wilson's disease	Dog	Adult tissue (surgical resection, needle biopsy, fine needle aspiration)	Nantasanti *et al.*^63^; Kruitwagen *et al.*^64^
Wolman's disease	Human	iPSCs (fibroblast-derived)	Ouchi *et al.*^44^

ESC, embryonic stem cell; GEMM, genetically engineered mouse model; iPSC, induced pluripotent stem cell.

### Monogenic diseases

Monogenic liver diseases encompass a group of disorders caused by mutations in a single gene that result in hepatic dysfunction with or without systemic manifestations.^46^ Chronic parenchymal liver damage caused by monogenic diseases represents a significant risk factor for the development of hepatocellular carcinoma (HCC). Despite their heterogeneity, all diseases share a common pathophysiological framework that involves continuous rounds of hepatocyte death and regeneration followed by inflammation and eventually the development of fibrosis and cirrhosis. The use of research models that recapitulate the genetic background of the individual patient is of central importance for the study of monogenic liver disease because, despite being restricted to a single gene, the number of distinct mutations can exceed 100. For this reason, organoid models represent a valuable novel tool that fulfils this important criterion.

#### Alagille syndrome

The Notch signalling pathway is a highly conserved signalling pathway that regulates embryonic development of several organs including the liver.^47^ Loss of function mutations in *JAG1* and *NOTCH* lead to a condition called Alagille syndrome (ALGS) that mainly affects the liver, heart, vertebrae, face and eyes.^48^ About 90% of the cases are caused by *JAG1* mutations (ALGS type 1) while only 1% are caused by mutations in *NOTCH2* (ALGS type 2). The characteristic hepatic manifestations of ALGS are bile duct paucity and chronic cholestasis, owing to the essential role of Notch signalling in the differentiation and maturation of cholangiocytes from hepatoblasts during embryonic liver development. Impaired biliary differentiation could be recapitulated in liver organoids derived from ALGS patients or mouse models.^24^^,^^49^ In healthy organoids, differentiation towards the biliary fate typically results in the upregulation of characteristic ductal markers such as KRT7 and KRT19. However, following differentiation, KRT19-positive cells in organoids derived from patients with ALGS were very low in number and predisposed to undergo apoptosis.^24^ Liver organoids derived from iPSCs represent alternative models for the study of Notch pathway deregulation^25^ or ALGS.^40^ In the elegant study by Guan and colleagues,^40^ iPSC technology combined with CRISPR/Cas9-based genome editing was used to introduce or reverse ALGS-causing mutations in healthy or disease liver organoids respectively. This approach enabled the pathogenicity of distinct *JAG1* mutations to be characterised in a patient-specific manner.

#### Alpha-1 antitrypsin deficiency

A1AT encoded by the *SERPINA1* gene, is the most common circulating serine protease inhibitor. A1AT is mainly produced by hepatocytes and functions as a crucial regulator of neutrophil elastase activity, protecting against proteolytic damage.^50^ A1AT deficiency is characterised by misfolding and accumulation of mutant protein in the endoplasmic reticulum (ER), that triggers ER stress and activates apoptosis. The most common mutation is caused by a single-nucleotide polymorphism resulting in a missense mutation at residue 342 (Glu342Lys), called the Z genotype. Homozygosity for the Z allele results in the most severe clinical manifestations of A1AT deficiency, with reduced A1AT secretion and plasma levels causing emphysema. Using biopsies from patients with homozygous *A1AT* mutants to derive liver organoid cultures, Huch and colleagues successfully reproduced the hallmarks of A1AT deficiency *in vitro*.^24^ Differentiation of chol-orgs into the hepatocyte lineage resulted in A1AT protein aggregates comparable to those found in the corresponding patient biopsies, and the reduced level of A1AT secreted in the culture supernatant was associated with a reduced ability to inhibit elastase function *in vitro*. Notably, differentiated organoids also recapitulated other disease phenotypes such as ER stress and increased apoptosis.^24^ In addition, a recent study reported the use of liver organoids derived from patients with different A1AT deficiency-causing genotypes.^51^ Remarkably, liver organoids reflect genotype-specific features observed in patients and provide a new system for validating mutations in rare genetic diseases.

#### Citrullinemia type 1

Citrullinemia type 1 (CTLN1), also known as Arginosuccinate Synthetase Deficiency, is a genetic disease caused by mutations in the enzyme Arginosuccinate synthetase (ASS1).^52^ ASS1 is a central enzyme of the urea cycle and essential for the conversion of excess ammonia into urea. Mutations in ASS1 impair its detoxifying function and result in severe symptoms caused by hyperammonemia. In contrast to other monogenic liver diseases, CTLN1 does not lead to parenchymal damage in the liver. Indeed, liver organoids derived from patients with CTLN1 had the same differentiation potential and morphological characteristics as those derived from healthy controls.^38^ Functional features such as albumin secretion, glycogen storage and lipid uptake were also indistinguishable. However, CTLN1 organoids clearly showed increased accumulation of ammonia that could be reversed by overexpression of ASS1. This proof-of-principle study for the rescue of impaired ammonia detoxification in liver organoids through genetic manipulation provides early evidence for future gene correction therapies combined with patient-derived organoid-based therapies.

#### Cystic fibrosis

Cystic fibrosis is the most common genetic disease affecting Caucasians.^53^ The underlying cause of the disease is a deregulation of epithelial fluid transport due to mutations in the gene encoding the chloride channel Cystic Fibrosis Transmembrane Conductance Regulator (CFTR). Mortality primarily results from thick tenacious mucus in the lungs that causes inflammation and recurrent infections. However, cystic fibrosis is a multisystemic disease and also affects other epithelial tissues of liver, pancreas, kidney, intestine and reproductive system. Deletion of phenylalanine at position 508 (F508del) is the most common mutation, yet more than 2,000 distinct mutations have been identified to date. Liver disease in cystic fibrosis is characterised by decreased alkalinity and fluidity of bile leading to biliary cirrhosis with or without portal hypertension.^54^ CFTR is expressed at the apical membrane of cholangiocytes and responds to hormone stimulation by increasing the intracellular cAMP level that mediates the efflux of chloride ions into the bile duct lumen. This process is impaired in individuals with mutant CFTR and can be modelled using organoids derived from patients with mutated CFTR.^55^ Organoids treated with forskolin, an adenylyl cyclase agonist that raises intracellular cAMP levels, respond by actively pumping fluid into their lumen resulting in visible swelling. Intestinal organoids derived from patients with cystic fibrosis fail to swell following forskolin treatment. The same phenotype could be reproduced in iPSC-derived CFTR-mutant chol-orgs.^25^^,^^26^ The surprisingly simple swelling assay offers the possibility to efficiently screen chemical modulators of CFTR function in a fast and personalised approach.^56^

#### Wilson's disease

Wilson's disease is a rare genetic condition that is primarily characterised by hepatic and neurologic symptoms resulting from abnormal copper accumulation in the liver and brain.^57^ Like many other metabolic processes, copper metabolism takes place in the hepatocyte. Copper is taken up in the hepatocyte through copper-specific transporters, before being delivered to ATP7B, a copper-dependent ATPase, which transfers it to the plasma carrier caeruloplasmin in the ER or excretes it into the bile (in cases of copper overload). Wilson's disease is caused by mutations in *ATP7B* that impair its function and result in intracellular copper accumulation. Copper overload causes hepatocyte death and uncontrolled release of copper in the circulation. Persistent hepatocyte damage and resulting chronic hepatitis predisposes patients to the development of cirrhosis and HCC. Like humans, dogs can be affected by copper-storage disease with similar manifestations as Wilson's disease.^58^ The underlying molecular origin of canine copper-storage disease is due to mutations in the scaffolding protein COMMD1 (also known as MURR1) that plays a critical role in the excretion of copper into the bile.^59^ So far, only little attention has been given to the fact that organoid technology has also been successfully applied to species other than humans and mice. Remarkably, organoid models of different tissues have been derived from dog, cat, cow, pig, horse, sheep, chicken and more recently from snakes.[60], [61], [62] In an attempt to model a copper-storage disease observed in dogs, Nantasanti and colleagues used canine liver organoids derived from dogs (with either wild-type or deficient *COMMD1* gene) to recapitulate features of the disease.^63^ Following treatment with CuCl_2_, *COMMD1*-deficient liver organoids accumulated more copper than wild-type controls and underwent cell death within 24 hours. Both phenotypes could be reverted by transduction and overexpression of a functional COMMD1 gene copy.^63^ In a recent follow-up study, the same authors provided preclinical proof for organoid-based cell transplantations.^64^ Liver organoids derived from *COMMD1*-deficient dogs were transduced with gene constructs encoding functional COMMD1 and then re-transplanted via the portal vein. Notably, despite low engraftment and proliferation rates *in vivo*, transplanted organoids persisted for up to 2 years, providing the first evidence for the safety of autologous liver organoid transplantations.^64^

#### Wolman's disease

Lysosomal acid lipase deficiency, also termed Wolman's disease, is a rare condition characterised by the accumulation of lipids due to reduced or absent function of the enzyme Lysosomal Acid Lipase (LIPA).^65^ Mutations in *LIPA* result in a dysfunctional enzymatic breakdown of triglycerides leading to hepatomegaly and hepatic failure. The disease shares many characteristics of fatty liver disease but can already manifest during infancy and is lethal if untreated. There is currently no consensus guideline for the treatment of patients affected by Wolman's disease, thus new effective therapies are urgently needed. Ouchi and colleagues, used an iPSC-derived liver organoid model of steatohepatitis and observed increased accumulation of lipids in organoids derived from patients with dysfunctional LIPA compared to healthy controls.^44^ The *in vitro* disease model was further exploited to test drugs with the efficiency to reverse the phenotype of lipid accumulation, providing a personalised system for drug discovery.

### Alcohol-related liver disease

Alcohol-related liver disease (ALD) is a highly prevalent chronic liver disease worldwide caused by chronic excessive alcohol consumption. The disease course typically involves the development of alcohol-related fatty liver, its progression to alcohol-related steatohepatitis and finally cirrhosis, that in a minority of patients culminates in the development of HCC.^66^ Cessation of alcohol consumption is the most important factor for successful therapy, which may involve anti-inflammatory treatments or liver transplantation in the most severe cases. Recently, Wang and colleagues developed an *in vitro* organoid model system that recapitulates typical features of ALD pathophysiology.^67^ ESC-derived liver organoids were co-cultured with foetal liver mesenchymal cells and treated with ethanol. Remarkably, compared to untreated controls, ethanol-treated organoids displayed several features of alcohol-induced liver injury such as increased CYP2E1 and CYP3A4 activity, oxidative stress, deposition of extracellular matrix, and apoptosis. It is conceivable that the reported co-culture system could be further exploited to model ASH by complementation with immune cells.

### Non-alcoholic fatty liver disease

NAFLD encompasses a range of conditions with varying degrees of parenchymal liver damage typically developing in patients with the metabolic syndrome. The disease ranges from hepatic steatosis, also termed non-alcoholic fatty liver (NAFL), to non-alcoholic steatohepatitis (NASH), a more severe condition that includes inflammation and hepatocyte damage and can progress to cirrhosis.^68^ As a consequence, these patients are also at high risk of developing HCC. The global prevalence of NAFLD is expected to rise and is predicted to significantly contribute to the rising incidence of NAFLD-related HCCs by 2030.^69^ Current human *in vitro* models for the study of NAFLD are based on PHHs and hepatoma cell lines,^70^ but the related shortcomings limit their use for personalised research. Ouchi and colleagues used a multicellular iPSC-derived organoid model to recapitulate key features of steatosis and steatohepatitis *in vitro*.^44^ Treatment of liver organoids with increasing doses of free fatty acids (FFAs) resulted in a gradual accumulation of intracellular lipids. This effect was paralleled by the secretion of inflammatory cytokines such as tumour necrosis factor-α, interleukin (IL)-6 and IL-8 by the Kupffer-like cells within the tri-lineage organoid. Moreover, prolonged treatment with FFA induced ballooning of the hepatocyte-like cells, upregulation of vimentin and α-smooth muscle actin expression, and the deposition of collagen, recapitulating key hallmarks of steatohepatitis. The levels of vitamin A in the stellate-like cell population decreased following FFA treatment, suggesting that they were activated. Remarkably, histological fibrosis resulting from FFA treatment, correlated with increasing stiffness of the liver organoids measured by atomic force microscopy. In a similar fashion, adult tissue-derived chol-orgs of human, mouse, cat, and dog origin accumulated lipids following treatment with FFA.^71^ A follow-up study assessed the feasibility of using the feline liver organoid system to screen drugs that reduce the accumulation of lipids and identified 2 promising candidates for further clinical evaluation.^72^ In conclusion, given the multiple cell types involved in the development of NAFLD and NASH, the ideal liver organoid system for studying the disease should be based on multilineage models, as described by Ouchi and colleagues,^44^ or perhaps even better, a co-culture system based on adult tissue-derived hep-orgs, stellate- and Kupffer cells.

### Primary liver cancer

Primary liver cancer is a major cause of cancer-related deaths worldwide. The disease affects over 1 million people every year and 830,000 die as a consequence.^73^ Most primary liver cancers are HCCs (75–85%), or intrahepatic cholangiocarcinomas (CCAs) (10–15%). In most countries, the incidence of HCC has more than doubled in the past 25 years and is predicted to rise significantly until 2030.^69^ As HCC develops predominantly in the context of chronic liver disease in patients with cirrhosis, it can be expected that a better control of the underlying risk factors, mostly HBV and HCV, will result in a decline in HCC incidence rates in high-risk countries such as China, Japan and Singapore.^69^ On the other hand, emerging non-viral risk factors such as NAFLD and its most severe form NASH will increase in importance particularly in Western Europe and the United States. The high mortality rate associated with HCC is linked to the fact that most patients are diagnosed at an advanced disease stage, by which point treatment options are limited.^74^ During the last decade, efforts to find new HCC therapies were mostly unsuccessful as witnessed by the large number of clinical trials that failed in phase III.^75^ Clearly, new therapeutic options are urgently needed.

A major obstacle for the development of new therapies and biomarkers for HCC has been the lack of appropriate *in vivo* and *in vitro* models that reflect the biology and heterogeneity of HCCs observed in patients. For decades, a limited number of 2D-grown cancer cell lines derived from hepatomas and HCCs have represented the state-of-the-art *in vitro* model for the study of HCC.^76^ Despite their broad utility, they suffer from significant shortcomings such as the lack of 3D growth and the absence of genetic heterogeneity due to their typical monoclonal nature. However, HCCs are characterised by a large degree of intratumour and interpatient genetic and phenotypic heterogeneity, and it is not clear how well cancer cell lines represent the tumour biology of HCC.^77^ A better representation of human HCC features could be achieved with the generation of patient-derived xenograft (PDX) models following transplantation of HCC tissue into immunodeficient mice.^78^ These models offer great advantages as they preserve the genetic and histologic features of the primary tumour as well as tumour-stroma interactions, making them promising tools for preclinical drug development and evaluation. However, establishing and maintaining PDX models is time- and labour-intensive, costly and eventually the models are not amenable to high-throughput compound screenings. Moreover, studies of the interaction between tumour and immune cells require additional efforts to generate humanised PDX models with a fully competent human immune system.^79^

The organoid technology could overcome limitations of cancer cell lines and PDX models because it combines the advantages of both systems. Indeed, the generation of patient-derived cancer organoids has been a major breakthrough in cancer biology. Over the past 5 years, tumour-derived organoids have been described for most organs for which healthy tissue organoids have already been generated.^80^ The ability to culture diseased and healthy tissue from the same patient is a major advantage over cancer cell lines and PDX models as it allows for the study of tumour development and progression, as well as providing patient-matched controls for drug testing assays.

**Fig. 2:**
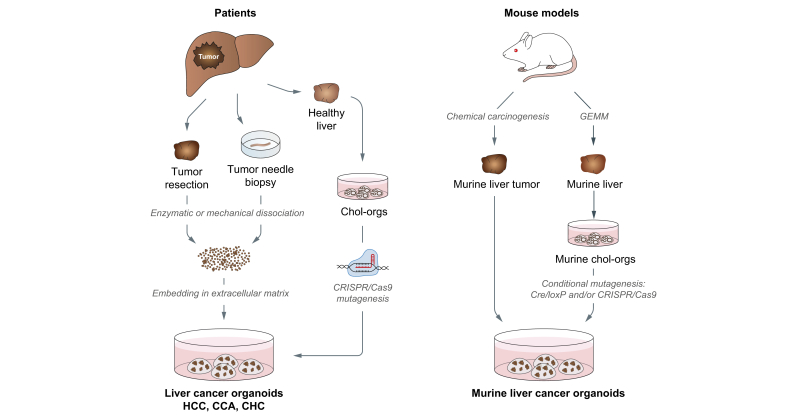
Workflow for the generation of liver cancer organoids. Liver cancer organoids can be directly established from patient tumour tissue collected after surgical resection or needle biopsy. The generation of cancer organoids follows the same protocol as described for normal liver organoids. Alternatively, liver cancer organoids can also be established from healthy chol-orgs using CRISPR/Cas9-mediated genetic engineering for the precise introduction of cancer driver mutations. Murine liver tumours derived from mouse models of chemical carcinogenesis can be used for the generation of murine liver cancer organoids. Genetically engineered mouse models carrying floxed oncogenes and/or tumour suppressors can be used to generate a flexible system based on conditionally mutant chol-orgs. CCA, cholangiocellular carcinoma; CHC, combined hepato-cholangiocarcinoma; CRISPR/Cas9, clustered regularly interspaced short palindromic repeats/CRISPR associated protein 9; GEMM, genetically engineered mouse model; HCC, hepatocellular carcinoma.

Combining the knowledge gained from growing healthy liver-derived organoids and cancer organoids from gastrointestinal cancers has allowed scientists to define culture protocols for the derivation of liver cancer-derived organoids from patients^81^^,^^82^ and chemically induced murine liver cancers^83^ (Fig. 2). Using surgical resection tissue, Broutier and colleagues established cancer organoids derived from patients with HCC, CCA and combined hepato-cholangiocarcinoma (CHC).^81^ Surgical resection, however, introduces a bias towards a minority of patients with early stage HCC^84^ and therefore does not include the whole disease spectrum. HCC and CCA organoids can also be generated from needle biopsy-derived tumour tissue, a strategy that can cover all HCC tumour stages including patients with advanced HCC who are typically treated with systemic therapies and most urgently require new treatment options.^82^ Irrespective of the tissue collection protocol, the derivation efficiency of HCC organoids was relatively low in both studies with an efficiency rate of ∼26% for biopsy-derived organoids and ∼27% for HCC organoids derived from surgical resections.^81^^,^^82^ Indeed, cancer organoid cultures could only be established from moderately to poorly differentiated HCCs (or grade III and IV according to Edmondson and Steiner^85^) that typically display a higher proliferative index compared to well differentiated tumours (Edmondson grade I and II). Importantly, no other clinical or histopathological parameter correlated with success or failure of HCC organoid generation. Notably, cancer organoids could be established from patients with all major underlying liver diseases and disease stages.

Histopathological diagnosis performed on biopsies and cancer organoids revealed that HCC and CCA organoids very closely recapitulated the histology of the primary tumours over long-term culture, indicating that the ability to reproduce the tumour phenotype *in vitro* is a tumour cell-intrinsic feature independent of other cell types present in the tumour microenvironment.^81^^,^^82^ Remarkably, whole exome sequencing analysis indicated that cancer organoids robustly preserved >90% of the genetic alterations found in the primary tumours and that only a small number of *de novo* mutations accumulated during long-term culture. The mutational landscape, predominantly characterised by recurrent mutations in *TP53*, *CTNNB1* and *ARID1A*, closely mirrored previously reported HCC and CCA cohorts. Of note, intratumour genetic heterogeneity, an important feature linked to tumour evolution and drug resistance, was preserved between cancer organoids and HCC biopsies. Cancer organoids also very closely preserved the gene expression profiles of the corresponding tumour tissues and were representative of a broad population of liver cancers from a TCGA cohort.^82^

A limitation of these organoid cultures is the lack of cell types other than epithelial or cancer cells. Indeed, stromal, endothelial and immune cells cannot be expanded simultaneously with cancer organoids using current protocols. However, co-culture systems can now be used to study the interactions between immune cells/cancer fibroblasts and tumour cells.[86], [87], [88]

Another important feature of cancer organoids is their tumorigenic potential *in vivo*. Subcutaneous xenotransplantation of liver cancer organoids into immunodeficient mice resulted in the growth of tumour xenografts with well-preserved histopathological features compared to the primary tumours. Interestingly, xenografts derived from CCA organoids displayed areas of desmoplastic stroma reaction typical of adenocarcinomas.^82^ Moreover, metastatic seeding of cancer cells in the lung of a patient with CCA could be recapitulated following the injection of the same patient's CCA organoids in the kidney capsule of an immunodeficient mouse.^81^ As previously discussed, cancer organoids enable similar throughput drug screening as cancer cell lines, but in a potentially more physiologically relevant context due to their superiority in retaining tumour-specific features. We and others tested the feasibility of using liver cancer organoids for *in vitro* drug treatments and found variable sensitivities to conventional chemotherapies and standard-of-care targeted therapies such as sorafenib.^81^^,^^82^^,^^89^ Finally, organoids and cancer organoids can be cryopreserved to generate living biobanks, providing a renewable resource of patient-derived tissue. These biobanks can be enriched with the patient's clinical history to include response to treatments, development of resistance, and survival.

Alternative approaches to derive cancer organoids for cancer modeling have also been explored. Using the CRISPR/Cas9 technology, normal intestinal organoids were transformed by sequential introduction of cancer driver gene mutations.^90^ Two recent studies reported the generation of organoid models based on chol-orgs that acquire tumourigenic features following genetic modification to incorporate mutations commonly found in CCAs.^91^^,^^92^ Remarkably, transplantation of such engineered organoids into immunodeficient mice gave rise to xenografts with carcinoma features with characteristics of either HCC or CCA depending on the inserted oncogenic driver. These approaches provide ideal models for probing the tumourigenic potential of individual oncogenes and tumour suppressors *in vitro* and *in vivo*.

### Primary sclerosing cholangitis

Primary sclerosing cholangitis (PSC) is a cholestatic liver disease characterised by chronic inflammation and fibrosis of the biliary tree.^93^ The underlying risk factors for the development of PSC are unknown, however, most patients with PSC also present with inflammatory bowel disease as a co-morbidity. The clinical management of PSC is unsatisfactory because, except for liver transplantation, there are no therapeutic options. The link with inflammatory bowel disease suggests an immune-mediated disorder but no pathophysiological mechanism has been identified. This is due in part to the fact that the available models for the study of cholangiocytes are limited. Human chol-orgs, however, are ideal *in vitro* models because of the high efficiency rate of generation and their preservation of genetic and morphologic characteristics of bile ducts.^24^^,^^27^ To better understand the molecular properties of cholangiocytes in PSC, Soker and colleagues established chol-org models from the bile collected from patients with PSC during diagnostic endoscopic retrograde cholangiopancreatography.^94^ Growth rate, expression of biliary markers and functional properties of PSC-derived organoids were comparable to chol-orgs derived from liver explants. Of note, RNAseq analysis of PSC organoids revealed the upregulation of genes related to immune regulation compared to non-PSC controls, suggesting that cholangiocytes derived from patients with PSC preserve the characteristics of the original *in vivo* tissue. In addition, following treatment with IL-17, bile-derived organoids actively secreted various chemokines and cytokines such as CCL20, LCN2, CXCL1 and S100A9. In conclusion, bile-derived organoids are promising models for the study of PSC pathophysiology and could be used to test compounds for the management of the inflammatory phenotype.

### Viral hepatitis

Despite major progress in prevention and treatment, viral hepatitis caused by infections with HBV/HCV remains a significant global health problem. HBV and HCV are both hepatotropic viruses and the receptors used for cell entry are well known. Thus, liver organoids could represent ideal cellular models for the study of host-virus interactions in a personalised manner. Indeed, liver organoids derived from iPSCs were susceptible to infection with HBV.^95^ Unlike hepatoma cell lines, iPSC-derived liver organoids endogenously expressed high levels of the HBV entry factor sodium-taurocholate cotransporting polypeptide (NTCP, encoded by *SLC10A1*). Various intracellular viral RNAs and DNAs could be successfully detected in organoids and culture supernatant, albeit at lower levels than in PHHs. In addition, the authors also infected 2D hepatocyte-like cells derived from the same donors and found a lower susceptibility to HBV compared to the organoids, suggesting that 3D growth might result in a more mature phenotype necessary for HBV infection. So far, no HBV or HCV infection models based on adult tissue-derived liver organoids have been reported. This is most likely because the current degree of hepatocyte differentiation achieved in liver organoids is insufficient to support the entire viral life cycle.

## Future perspectives

**Fig. 3:**
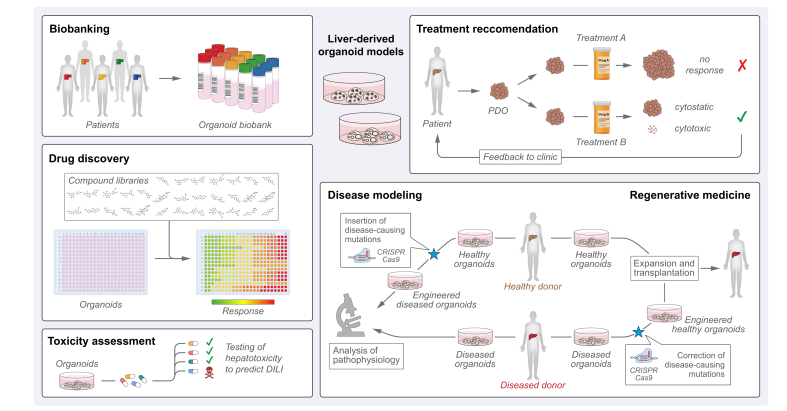
Translational applications of liver-derived organoid models. Patient-derived organoid biobanks are important resources for applications in academic research and drug development. Organoid models of liver disease can be directly generated from diseased donors or alternatively from healthy donors following CRISPR/Cas9-mediated insertion of disease-causing mutations, *e.g.* for the study of monogenic liver diseases or liver cancer. Organoid-based drug discovery allows for a more physiological assessment of drug sensitivity and hepatotoxicity, facilitating the selection of potent drugs with a safe profile. PDOs could support clinicians during the therapeutic decision-making process by predicting the efficacy of different treatments for the same indication. Finally, organoid-based cell therapies represent an alternative to liver transplantation for various diseases, in particular monogenic liver diseases that can be corrected with genome editing methods. CRISPR/Cas9, clustered regularly interspaced short palindromic repeats/CRISPR associated protein 9; DILI, drug-induced liver injury; PDO, patient-derived organoid.

Liver-derived organoid models are being rapidly integrated into various aspects of biomedical research and are continually evolving due to improved derivation protocols and culture conditions (Fig. 3). Despite the recent novelty of the technology, several liver disease models recapitulating the most important pathogenic features have already been established. Applying organoids to the study of liver diseases or drug screenings has the potential to greatly reduce the number of animal models used for equivalent purposes. In light of all the excitement around organoid technology, with all its different advantages (Box 1), there are still significant limitations. First, the classic hep-org and chol-org derivation protocols, as well as most organoid culture systems from various tissues, are designed to maintain and expand epithelial cells and thus lack other cell types that are typically present in their respective organs. This reduction in cellular complexity clearly limits the usefulness of organoids for the study of complex pathophysiologic processes such as fibrosis, hepatitis and liver cancer, where several different cell types interact in a highly dynamic way. Improved versions of organoid culture protocols try to address these limitations using either systems that incorporate and allow the parallel culture and differentiation of various cell types such as stellate-like cells, Kupffer-like cells, and endothelial cells^36^^,^^37^^,^^44^^,^^67^ or using co-culture protocols that re-introduce tissue-derived or engineered cell populations such as immune cells^87^^,^^88^^,^[96], [97], [98] and cancer-associated fibroblasts^86^ for cancer organoid cultures. Remarkably, a recent report described a protocol for the culture of patient-derived glioblastoma organoids that preserve and continuously generate diverse cell types, therefore maintaining a nearly intact tumour microenvironment at the cellular level.^99^ Whether such an approach could be applied to chol-orgs/hep-orgs or liver cancer organoids remains to be explored. Second and most likely of highest interest considering potential applications in the setting of regenerative medicine, the protocols to differentiate chol-orgs into hepatocyte-like cells could benefit from further improvement to increase the hepatic maturation level. Likewise, modifications to the culture conditions can also be expected to improve the derivation and long-term growth of adult liver-derived hep-orgs. Both objectives could possibly be reached using co-cultures with cell types that support the maturation of hepatocytes. Moreover, increasing availability of fresh tissue will be central for the generation of liver cancer organoids, particularly because of the scarcity of fresh tumour tissue from patients with advanced HCC. Finally, cellular heterogeneity in normal and diseased tissue will have to be addressed in more sophisticated co-culture models to better understand the pathophysiology of liver diseases and support the development of novel therapies.

Box 1 **Liver organoid systems: current advantages, limitations and potential solutions.**

**Figure:**
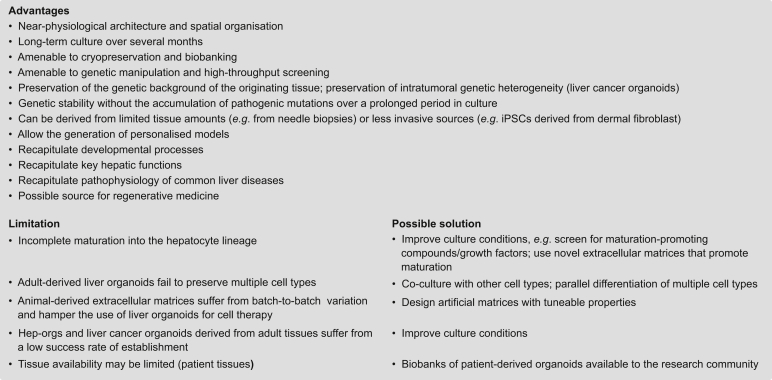

iPSCs, induced pluripotent stem cells.

## Financial support

M.H.H. is supported by a European Research Council Synergy grant 609883 (MERiC), by 10.13039/501100012390SystemsX.ch grant MERiC and by the 10.13039/501100001711Swiss National Science Foundation grant 310030B_185371.

## Author's contributions

Both authors contributed equally to the production of this manuscript.

## Conflict of interest

The authors declare no conflicts of interest related to this work.

Please refer to the accompanying ICMJE disclosure forms for further details.
